# Nanomembranes as Eco-Friendly Instruments for Modern Food Processing, from Filtration to Packaging

**DOI:** 10.3390/membranes15060167

**Published:** 2025-06-02

**Authors:** Simona Gavrilaș

**Affiliations:** Faculty of Food Engineering, Tourism and Environmental Protection, “Aurel Vlaicu” University of Arad, 2-4 E. Drăgoi Str., 310330 Arad, Romania; simona.gavrilas@uav.ro

**Keywords:** food waste valorization, contaminants withdraw, environmental impact, preservation, thin layer

## Abstract

With the increasing demand for safe, high-quality, and sustainable food, nanomembranes have attracted significant interest as innovative solutions in food processing. They are extremely thin structures created from special materials that allow for the selective filtration of very small particles. In the food industry, such approaches are increasingly used for packaging and processing, as they can slow down food degradation and thus extend its shelf life. This article examines the potential of utilizing nanomembranes as ecological tools at various stages of the food chain, ranging from advanced filtration of food liquids to the development of smart and active packaging. This study reviews the recent research in the field, highlighting the applications developed and presenting targeted advantages and disadvantages. The developed applications primarily focus on extending the shelf life of products while also discussing their antioxidant and antibacterial attributes. By highlighting the latest applications and emerging research directions, this article underscores the pivotal role of nanomembranes in facilitating the transition to a modern, sustainable, and environmentally responsible food industry. However, current research faces several challenges. Most products are less biodegradable and, consequently, could harm the environment. Additionally, data on the long-term effects of these materials on human health, particularly when used in packaging that comes into direct contact with food, remain insufficient. Therefore, more sustainable solutions are needed, such as nanomembranes based on natural biopolymers. Further studies are required to assess their safety and real-world effectiveness under industrial conditions.

## 1. Introduction

### 1.1. Sustainability Background

A priority for increasing the cost-effectiveness of technological processes is designing low-impact technologies. These technologies ensure sustainability through innovative solutions that minimize the negative effects on nature and reduce resource consumption. They play a key role in addressing the challenges of climate change [1], pollution, and the overuse of supplies. Several actors, such as educators, intermediaries [2], entrepreneurs, and local and national authorities, must jointly achieve the main proposed objectives. Various techniques have been evaluated and successfully implemented in this direction. In this sense, innovations in renewable energy sources and efficiency technologies, as well as sustainable agriculture, materials, transportation systems, waste management, carbon capture and storage, and water technologies, could be considered. The degree of practical implementation varies globally. Several examples of good practices include protecting cities against the effects of climate change [3] and implementing various waste treatment strategies [1,4].

This review highlights the current literature concerning the benefits of using nanomembrane techniques to limit the effects of various activities on different ecosystems. Nanomembrane techniques play a key role in developing green and sustainable solutions, have diverse applications, and directly contribute to reducing environmental impacts. Such efficient and versatile technologies often consume less energy than conventional alternatives. A key characteristic of these techniques is the absence of phase modification [5]. Their application in water and wastewater treatment [6,7,8,9], energy efficiency [10,11,12], agriculture sustainability [13,14,15], separations [16], and waste management supports their integration into low-environmental-impact technologies. Salehi discussed the potential benefits of using nanomembranes in dairy and sugar processing [17]. The qualitative wastewater parameters that result from integrating such devices into the technological flow must be considered [18] to support the environmental benefits of their implementation.

This study examines recent advanced applications in food processing from two primary perspectives. The first encompasses the general types of nanomembranes, their advantages, and their environmental risks. The second examines the relevance of membrane-based technologies in the food industry. Their widespread implementation may support a transition to more environmentally friendly practices, protecting the environment and reducing the impact of such manufacturing activities on natural resources. The originality of this work lies in its emphasis on specific field applications. The choice of this domain was motivated by a desire to raise awareness of durable and secure nano products that have undergone quantitative and, most importantly, qualitative validation, particularly as global needs increase.

### 1.2. Distinct Approaches for Membranes

In the context of global challenges related to climate change, population growth, and the depletion of natural resources, the food industry is facing increasing pressure to adopt sustainable and efficient solutions. One of the most promising technological directions that addresses these needs is the use of nanomembranes. These are ultra-thin materials with extremely precise filtration and separation properties. Their nanometric structure enables the fine control of substance transfer, opening up new perspectives in purification, nutrient recovery, food preservation [19], and waste reduction. Integrating them into food production chains optimizes water [20] and energy consumption, increasing product safety and quality and thus providing a sustainable development model for the future of the food industry [21,22,23].

Nanomembranes perform essential functions in two key areas of food processing: filtration and packaging. Although both contribute to product safety, quality, and sustainability, their roles differ in purpose, functionality, and technological implementation.

The nanomembranes used in filtration, purification, separation, and concentration selectively separate molecules or particles from a liquid or gaseous mixture without using heat or chemical additives. They act as semipermeable barriers, allowing for the passage of molecules (usually water, ions, or small organic compounds) while retaining larger particles such as bacteria, proteins, complex sugars, or fats. Several applications have been developed for the dairy industry (to develop lactose-free products) [24,25], for wine and beer processing (to remove microorganisms such as malolactic bacteria without pasteurization) to maintain a product’s original flavor and composition [26,27], to decrease ethanol levels [28,29,30,31] or sugar levels in juice processing [32], and to produce drinking water (via treating raw water to remove biological or chemical contaminants before use in processing). A membrane’s ability to allow the passage of certain molecules while blocking others, depending on size, electrical charge, or polarity, characterizes its molecular selectivity.

Nanomembranes are used in packaging for protection, atmospheric control, and shelf-life extension. Their main role is to control gas, humidity, and exchanges between the food product and the external environment, as well as microorganism development or contamination, to maintain the freshness and safety of the product. Their sustained use in this direction is based on their ability to exhibit selective permeability. This attribute represents the extent to which a membrane allows certain substances to pass through. This is crucial in filtration and controlled atmosphere packaging processes.

Integrating nanomembranes into packaging enables the selective control of oxygen, carbon dioxide, and water vapor, as well as the gradual release of compounds of interest (e.g., antimicrobials or antioxidants). This type of packaging is known as active or intelligent packaging, and its characteristics stem from its functionalization. The surface is chemically modified through this procedure to acquire specific properties, such as antimicrobial activity, hydrophobicity, or selective affinity.

Nanomembranes that regulate oxygen levels to prevent oxidation and bacterial growth without altering the product’s color could be considered for fresh meat packaging [33,34]. In fruit and vegetable packaging, atmospheric control through the membrane enables decreased oxygen and increased carbon dioxide levels, thereby slowing down ripening and reducing post-harvest losses [35]. Nanomembranes functionalized with silver nanoparticles or natural extracts could inhibit mold growth on packaged cheese during storage [36,37]. Two mechanisms could transform the membrane into an active type. One supposes interaction with the product and the other with the external environment. The outcome reflects extending the product’s shelf life by absorbing oxygen or releasing natural preservatives.

Using these nanomembranes reduces energy consumption, eliminates the need for chemical preservatives, and improves the quality of the final product [38]. They also extend shelf life, reduce food waste, and eliminate the need for artificial preservatives.

The roles of such tools in food processing are complementary, but there is a common goal. While nanomembrane filtration is utilized in the food processing phase to ensure efficient purification and separation of components, nanomembrane packaging is employed in the post-processing phase, providing protection to the final product and enhancing its durability. Together, these two applications contribute decisively to creating a safer, more efficient, and more sustainable food system.

## 2. Nanomembranes

### 2.1. Background

**Figure 1:**
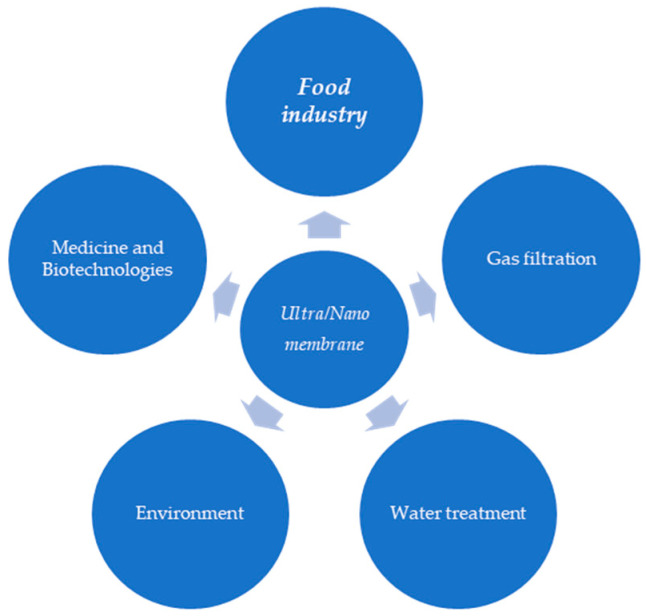
Main fields of application of membranes.

The observation of biological systems formed the basis for incorporating membrane techniques into different industrial processes. Membranes are characterized by the capacity to allow various molecules to cross the separation environment based on their specific features [39] and the aim of the process. An important attribute of these instruments is their capacity to be recycled [40], an aspect currently studied in industrial design processes. Figure 1 shows some of the possible links between the nanomembrane types and their fields of application.

As mentioned above, nanomembranes are nanometer-thick structures with precisely controlled morphological, chemical, and functional characteristics. This ultra-thin layer, made of polymeric, ceramic, metallic, or composite materials, offers remarkable selectivity, permeability, and chemical stability. Thanks to these qualities, nanomembranes are being increasingly used in various fields, including water purification, pharmaceuticals, regenerative medicine, microelectronics, and, most notably, the food industry. They can be considered an innovative solution for enhancing product quality, improving process efficiency, and promoting environmental sustainability.

**Figure 2:**
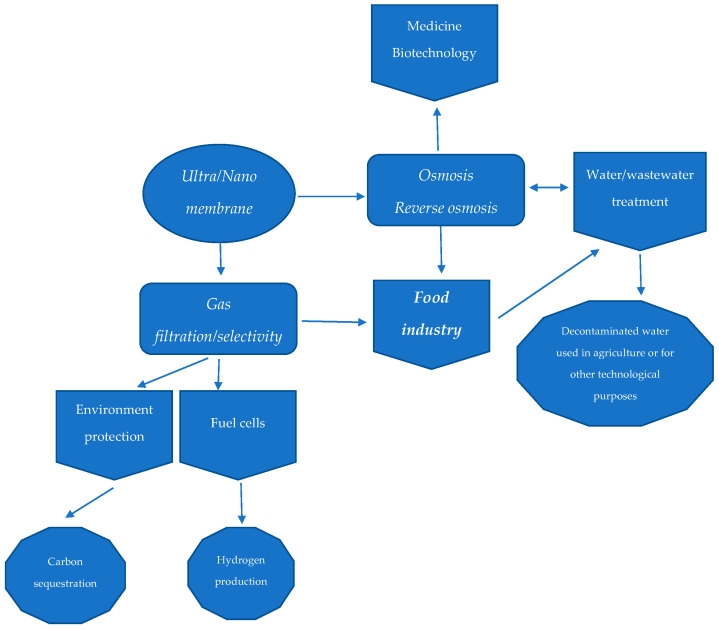
Links between nanomembrane functions and areas of implementation.

The diverse areas in which these devices can be used highlight their versatility. Figure 2 illustrates the commonalities between the membrane domains of application and the primary functions that support their use.

As mentioned above, membranes play a significant role in water and wastewater treatment. These areas have a considerable impact on human health and security, as nanofiltration is a separation process that utilizes membranes with nanometer-sized pores (1–10 nm) to remove small organic molecules, divalent salts, and microorganisms. This can provide more fresh water for the population, given that natural resources have recently decreased. Related to this, the possibility of sterilizing filtration should be mentioned, particularly in the context of ultra- and nanofiltration, where the removal of small particles, such as organic molecules or viruses, can be ensured. This technology has significant applications in food processing. This consideration is supported by the need to achieve market growth and meet public demand for less processed products, particularly in light of the thermal processes required to limit microbial growth. Under specific conditions, aseptic conditions can be achieved through membrane filtration, with process efficiency depending on the membrane and filtrate characteristics. There must be a correlation between the layer’s characteristics, such as the porous diameter and the filter temperature, which could significantly influence the process yield [41].

Through wastewater management, recovered sewer water can be used for various technological or irrigation purposes, thereby reducing pressure on the water supply. Wastewater treatment can reduce levels of heavy metals and microorganisms. In addition to the previously mentioned areas, a significant advancement in the wastewater field could be the development of efficient mechanisms to separate the liquid mixtures with components of different densities. This could include effluents from meat, oil, and dairy industry processing, where a certain quantity of greasy compounds may be encountered. A possible solution was presented by He et al., who tested the separation and antibiotic efficiencies of polyvinylidene fluoride electrospun nanofibrous felt covered with polydopamine and TiO_2_ nanoparticles [42].

Nanomembrane application in seawater desalination is also worth investigating to understand its positive impact on water remediation better. This technology is considered eco-friendly since it consumes less energy than the classical approach.

As Figure 2 suggests, there are common goals in developing nanomembrane applications in the food and health sectors regarding specific safeguard attributes, reliability, and performance. As Zhang et al. mentioned, several formulas have been developed, and their performance continues to improve. Finding an appropriate formula to ensure the increased innocuity of the process with multiple uses remains a considerable challenge [43]. Tahir et al. tested the bactericidal and antioxidant properties exhibited by a PVA appliance with added cucumber and aloe vera extracts for skin problems [44].

A common benefit of using membrane techniques for separation is their ability to separate particles of different sizes. Studies have considered material fractions characterized by dimensions in the order of μm. The individual scale of the targeted molecule indicates the type of process to use. Reverse osmosis (RO) [45], which involves passing a solvent through a porous surface opposite the natural direction by applying hydrostatic pressure that exceeds the osmotic pressure, could be suitable for molecules whose dimensions reach 0.1 nm. In the case of nanofiltration (NF), this unit range could reach 2 × 10^−3^ μm. Nanomembrane techniques can be applied in biotechnologies, medicine, and food to target both organic and inorganic molecules. Comparing the information presented, we can recommend reverse osmosis for larger molecules, such as salts with higher valence metal ions, and the use of nanofiltration for monovalent ones. Many applications have been developed using polymer and fine film-type layers. Good performance can be achieved by incorporating organic and inorganic nanomolecules into the membranes. Their main disadvantage is the higher price than the classical types [46]. Geng et al. suggested applying NF based on the slight layer to remove micro- and nano-synthetic composites [47]. Such an application could have a significant impact on human and environmental safety levels.

Existing data in the literature highlight the tendency to implement nanofiltration to the detriment of reverse osmosis. This is due to the increased yield and reduced power of the filtration procedure [48]. Sawadogo et al.’s research supports the advantages of incorporating RO or NF into the standard sugar cane wastewater management protocol. Their approach efficiently increased pigment and removed inorganic molecules, achieving almost total elimination (98–99%) [49].

Nano-range materials are often substituted for traditional options in various applications. As suggested in Figure 2, ultra-thin layers could be used in gas separation procedures. Hazarika and Igole highlight the potential benefits of utilizing these tools in this domain [50].

Nanomembranes represent a cutting-edge technology with revolutionary potential in the food industry. Through their ability to achieve precise separation at the molecular scale, these structures contribute to the development of cleaner, more efficient, and more sustainable processes. Integrating nanomembranes into food production processes is timely and globally essential, as the industry faces increasingly stringent requirements regarding food safety, waste reduction, and energy efficiency.

### 2.2. Nanomembrane Classification

#### 2.2.1. Carbon Type

Carbon nanomembranes are considered advanced materials as they are produced via nanotechnology. Remy et al. highlighted the common features of membranes containing carbon nanoparticles. An aspect that must be highlighted and could contribute to the food sector is the synergy of such products for the construction and operational filling of particles [51]. They can be regarded as mimetic products [52] since natural membranes serve as the starting point for their design, having nano-thickness dimensions and high selectivity. Dimitropoulos et al. indicated a direct dependency between the layer’s width and the mechanical properties exhibited. Separation efficiency could be linked to the material’s pore characteristics [53]. For multiple coatings, the composite performance depends on the individual membrane features [54].

Carbon nanomembranes exhibit varying performance levels and have multiple applications due to the diverse possible carbon configurations, including graphene, diamond, carbon nanotubes (CNTs), nanostructured activated carbon, and carbon fibers [55]. Their specific characteristics support their use in various industrial applications due to carbon’s ability to form strong bonds [56]. Their strengths include their increased fine porosity, high mechanical strength, antibacterial properties, and the ability to filter and separate various particles at the nanoscale [57]. Such properties and individual nanostructuring highlight the possibility of their integration into advanced frameworks [55,57].

Some of a carbon nanomembrane’s characteristics, such as significant selective permeability, mechanical and chemical resistance, antibacterial properties, and high adsorption capacity, can be successfully utilized in various processes within the food industry. We could consider specific water filtration and purification applications for this domain, such as using raw components or for recovery and reuse purposes, smart food packaging, separating and refining different constituents, removing contaminants from fruits and oilseeds, and processing raw materials.

Graphene-based nanomembranes are used for desalinating and purifying water used in food production, effectively eliminating bacteria, viruses, and heavy metals. Applying such an approach could also have an ecological effect since it may contribute to reducing water consumption and operational costs. This is a domain in which nanomembranes are an effective alternative to traditional reverse osmosis technologies.

The above nanomembranes have been studied regarding their possible use as packaging elements. Their utilization could help extend the shelf life of food [58] by reducing exposure to moisture and oxygen and ensuring the product’s innocuity via their antibacterial characteristics. This application could be considered, especially for perishable foods, to prevent microbial growth. Specific safety tests, including the toxicological approach, are recommended before considering implementing such an approach.

An important aspect of nanomembranes with respect to their use in food products is their increased selectivity capacity. They can be used to obtain products with low levels of different biomolecules or to remove potentially harmful contaminants, such as pesticides, aflatoxins, and other toxins. Potential examples include foods with decreased or zero levels of lactose, alcohol, or proteins. Thus, sectors such as milk or beverage processing could benefit from implementing these instruments. Moreover, the quality and safety of the final product are improved by eliminating impurities that could be introduced during production. In this regard, we can consider sediments from the fermentation stages of wine or beer or the procedure applied to press or extract juice and oil. Another direction for successful use involves monitoring changes in various parameters. In this sense, we could consider the biochemical transformation of biomolecules during the fermentative (alcoholic or lactic) [59] or maturation processes. Accurate and rapid responses could be obtained using sensors with specific sensitive membranes. Table 1 presents some recent applications of carbon-type membranes in the food processing industry.

As presented above, there is increased interest in utilizing and designing carbon nanomembranes for improving water quality and as components of packaging composites. The proposed products would be composed of several layers of different materials (e.g., polymers and nanomaterials). Such a design, in the form of a composite, provides both mechanical strength and advanced functionality. These observations could also highlight the other advantages of these instruments, especially in the food sector processing. In addition to their contribution to reducing water pollution, which involves their integration into filtration, the use of nanomembranes could decrease chemical use by replacing traditional purification and sterilization processes, which require hazardous chemicals, with nanomembranes. Energy efficiency improvements could also be observed because nanomembrane-based processes require less power than other technologies, such as distillation or reverse osmosis. Furthermore, they could contribute to reducing food waste by extending the shelf life of food products. Several practical studies have already been conducted in this regard, as shown in Table 1.

This approach has several weaknesses, as it is still in its development phase. These issues could serve as a basis for future research strategies to mitigate their negative impact on the environment or human health. Some aspects that could determine future study directions concern the effects of nanomaterial waste. The production and disposal of nanomembranes may release nanoparticles into the environment with little understood long-term impacts. The approach also has high production costs; nanomembrane technology is still developing and more expensive than traditional methods. Its implementation could pose possible health risks because some nanoparticles may have toxic effects on living organisms; therefore, research into their safety is ongoing. Various particle migration processes from the packaging material to the food can occur due to the specific characteristics of the wrapped product, such as pH, lipophilicity, and wettability [66,67,68].

#### 2.2.2. Metal Type

Metal nanomembranes are advanced structures composed mainly of nanoscale metals or metal oxides. They have extremely fine pores that allow for the selective separation of particles and contaminants. Due to their filtration, catalysis, and antimicrobial protection properties, such instruments can be used in various industries, including the food industry.

The metals in these membranes possess specific properties that support their successful implementation in various food processing applications. Commonly incorporated metals are silver (Ag) and copper (Cu/CuO), which are used for their strong antimicrobial and purification properties, respectively, and gold (Au), which is used in sensing and catalysis applications. Titanium (Ti/TiO_2_) or aluminum (Al) are often used for filtration and purification. Bashmbu et al. mentioned the possible application of SiO_2_ to separate oil and water mixtures [69]. Their concept was based on the hydrophilic attributes of oxide-based membranes.

As can be seen, there are common features between carbon and metal-type nanomembranes. Both exhibit a high filtration capacity, removing impurities, microorganisms, and unwanted particles at the nanoscale. Differences also exist in that metal-containing nanomembranes can address some disadvantages of carbon nanomembranes. The possible limitations of carbon membranes in reaction media include high temperatures, extreme pH levels, and aggressive chemicals. Such deficiencies could be resolved using metal nanomembranes, which have proven chemical and mechanical resistance and stability. Their catalytic capacity also highlights their improved performance. These devices can accelerate chemical reactions and have proven useful in the decomposition of contaminants.

The properties mentioned above make metal nanomembranes viable and potentially useful in multiple areas of the food industry. The main fields of interest target an important resource used in food processing: water quality. From this perspective, the main attributes of metal nanomembranes are purification and filtration, especially for nanomembranes based on TiO_2_ and Ag. Their application removes organic impurities, bacteria, and viruses from water used in food production processes, desalinates water, and removes heavy metals from water, reducing freshwater consumption.

Using silver and copper nanoparticles in packaging layers helps to extend the shelf life of food, preventing the development of contaminating microorganisms. This attribute is determined by the two cations’ capacity to form a strong bond with the contaminant’s membrane and influence their denaturation [70].

Nanomembranes containing titanium dioxide (TiO_2_) could substantially contribute to essential aspects of food processing, mainly in the preservation area. These particles could be used in materials with photocatalytic properties, which reduce surface contamination and ensure better food preservation. They could also remove bacteria and chemical residues from the surfaces of fruits and vegetables. Table 2 summarizes some recent applications of carbon-type membranes in the food processing industry.

The presence of nanoparticles in the composite structure could imply a synergistic mechanism. In a study by Demirbas et al., an increased inhibition area was observed for all tested microorganisms. A lower yield was obtained for *S. aureus* (15%), and the highest yield was found for *A. hydrophila* (22.5%) [74].

Although the potential benefits of incorporating nanoparticles into a membrane structure have been explored in several studies, these studies also included recommendations for further research, particularly to establish a toxicological profile due to the potential migration of ions into the products [81]. Mohammad et al. suggested that there may be differences in toxicity between nanoparticles present in a food product and those at the surface [82]. The potential ecological impact of metal nanoparticles resulting from the production and use of these membranes must also be considered, as they could contaminate soil and water, potentially affecting living organisms. The entrance and persistence of metal nanoparticles in the human body or other organisms could determine their long-term toxic effects. This could even result in the form of possible microbial resistance due to the excessive use of antimicrobial nanoparticles, such as silver and copper. The economic aspect should not be neglected because producing such materials requires the use of advanced technologies, and high costs may make their large-scale implementation difficult.

#### 2.2.3. Polymer Type

Polymer nanomembranes are advanced materials made from synthetic or natural polymers with nanoscale porosity. These membranes are used to separate, filter [83], and purify various compounds [84]. They are essential in food industry processes due to their flexibility, low cost, and high performance.

The potential applications of various polymers in food processing have been demonstrated. Good performance has been shown for polyamides, polypropylene, polyethylene, and polyvinylidene fluoride in reverse osmosis, ultrafiltration, or, in some cases, packaging [85]. Attention should be paid to the use of biodegradable natural polymers, such as chitosan, alginate, starch, or cellulose, in eco-friendly packaging [86].

Polymer nanomembranes can be manufactured with customized dimensions and characteristics. These attributes, along with flexibility, adaptability, and high selectivity, offer numerous possibilities for their practical implementation. These polymer nanomembranes enable the efficient separation of particles, microorganisms, and contaminants. They have a lower production cost than the previously discussed types of nanomembranes. Their antimicrobial characteristics mainly result from the inclusion of polymers with cations such as silver and copper. Different nanoparticles or nano-infillers could increase mechanical and permeability properties [87].

As mentioned above, polymer nanomembranes have multiple potential applications in food industry processes due to their properties. In addition to filtration and reverse osmosis, a significant contribution is made regarding packaging. Chitosan- and cellulose-antimicrobial-functionalized smart materials could contribute to extending the shelf life of food [88]. Polyethylene films modified with nanoparticles may reduce oxygen and moisture permeability, protecting food from spoilage. The possibility of encapsulating and controlling the release of ingredients may be explored in functionalized foods to preserve sensitive components, such as vitamins, probiotics, and essential oils, ensuring their gradual release into food products [89]. Another field of biomimetic foods that could be explored is the potential use of nanomembranes for emulsification operations [90]. Table 3 summarizes some recent applications of polymer-type membranes in the food processing industry.

These polymer-based applications are primarily developed to target preservation and extend shelf life. This research direction prioritizes analyzing the potential of biocomponents. This approach aligns with this study’s focus on durability.

Long-term protection was observed not only in the foods analyzed but also for microorganisms. Wang et al.’s results present a viability maintenance study of encapsulated *Lacticaseibacillus rhamnosus* GG. After 28 days at 4 °C and 25 °C, the yield of the control sample was approximately 22% lower [91]. Xia et al. outlined the significant influence of product exposure temperature on the film’s compound-release capacity or water vapor permeability. The techniques used to obtain the film exhibit comparable characteristics [94].

Some advantages polymer nanomembranes could bring to food production relate to purification and recycling. Integrating silver and copper could increase product innocuity and reduce the use of artificial preservatives to prolong its freshness. By developing smart packaging and efficient filtration, food waste can be reduced, thereby increasing preservation time.

Lower production costs can be expected, especially when natural polymers are used, which can be obtained through the valorization of agro-waste. This will decrease the environmental impact through two mechanisms: using biodegradable packaging materials and reusing vegetable waste by integrating it as a raw material in a new technological process. Designing and implementing such products could provide eco-friendly solutions for some of the negative aspects of using polymer nanomembranes, most of which are not biodegradable and present difficulties for recycling, contributing to microplastic pollution. They can also release degradation compounds when in contact with foods with acidic pH values or when exposed to increased temperature.

To be used in the food industry, nanomembranes must meet stringent requirements for safety, efficiency, and compatibility with various types of food. Polymer membranes are particularly advantageous due to their design flexibility, low cost, ease of processing, and clear regulations. They offer an optimal balance between performance and affordability, making them the first choice in large-scale industrial applications.

#### 2.2.4. Zeolite Type

Zeolites are natural or synthetic aluminosilicates with a three-dimensional crystalline network of pores and channels of precise dimensions, enabling the selective adsorption of molecules based on their size and polarity. They can filter substances at the molecular scale thanks to their uniform pores. Their increased adsorption capacity makes them suitable for capturing and retaining water molecules, heavy metals, toxins, and odors. Considering these characteristics, such membranes could be used to remove micropollutants and pesticides from water used in food production. Membranes containing zeolites are included in this category because they meet certain key classification criteria relating to structure, pore size, and nanoscale operation. Layers enclosing zeolites are classified as nanomembranes because they have nanoscale pores, provide precise molecular selection, and operate at the nanoscale. Their application may be extended to eliminating dyes or grease [95] compounds from processing oilseeds or fruits and vegetables with tinctorial attributes, as well as from wastewater [96]. Such techniques could lead to successful vegetable oil refinement by analyzing the same fundamental principles. Removing impurities and water could improve the oil’s oxidative stability.

These materials’ catalytic [97] and antimicrobial properties could increase their potential applicability in food processing [98]. Sakai mentioned that their alcohol dehydration capacity presents possible applications in food processing [99].

The capacity of these compounds to accelerate substrate transformation under mild conditions is another attribute that could be explored. An antimicrobial synergetic outcome could be obtained by incorporating different ions, such as silver [100], copper, or zinc, into composites.

Zeolite nanomembranes can remove heavy metal ions, such as lead, mercury, and cadmium, and absorb ammonia and nitrates. Zhu et al. highlighted the potential use of 2D metal-organic or covalent–organic frameworks and zeolite nanosheets for aqueous-linked splitting [101].

Zeolites can be integrated into packaging materials to absorb moisture and harmful gases such as ethylene, accelerating fruit degradation. Zeolite-based packaging with silver or copper ions has antimicrobial properties, extending the shelf life of food. Sani et al. emphasized the feasibility of utilizing zeolitic imidazolate framework systems for designing sustainable food packaging materials [102].

Due to their similarity with the clay materials frequently used in the wine industry, their main function could be clarification [103]. They do not have a laminar structure, and they could be safely used in food processing since they are relatively inert [104], allowing them to be used at high temperatures and in the presence of aggressive chemicals. Another important derivative attribute of zeolites is the possibility of subjecting them to antiseptic treatment [105].

Four common classes of zeolites are used in nanomembranes, depending on the intended use. Zeolite A is frequently utilized in filtration and ion exchange processes. Zeolites X and Y could be used in water purification and metal-ion capture. The ZSM-5 zeolite is used in catalysis and for removing organic compounds. For food detoxification and gas filtration, mordenite and clinoptilolite are recommended.

Negishi et al. successfully tested the capacity of Na-ZSM-5 for orange, pineapple, and apple juice concentration [106]. In their study, cylindrical membranes were employed in an osmotic process. The membranes remained inert at increased temperatures and responded well to fouling over a five-day work period.

Considering the catalytic capacity of zeolites, their incorporation in membranes could be the basis for designing and implementing sustainable processes. Several studies have demonstrated the usefulness of absorbent capacity in oil remediation [107] or transformation [108] treatments. Such an application could contribute to food durability since the quantity of used oils resulting from specific operations may constitute a significant environmental pollutant.

Given the specific properties of zeolites, their incorporation into composite layers may benefit the development of sustainable technological operations. If natural and renewable mineral-based materials have a low environmental impact and their extraction or synthesis, their use could be explored in eco-friendly industrial processes. However, differences must be considered when their nature is analyzed. Certain natural zeolites are biodegradable and do not generate persistent environmental waste. On the other hand, there are a few disadvantages to their use. There could be difficulties in recycling since, after use, zeolites saturated with heavy metals require special treatment for disposal. Moreover, they may exhibit potential ecotoxicity at certain concentrations, potentially affecting human health if ingested in large quantities or entering natural ecosystems. The environment is more susceptible to their presence, especially in the case of nano-dimension zeolites.

From an economic perspective, native types may have low costs, while the cost of producing synthetic versions may be considerable. Sustainability analysis must balance both the financial and technological aspects.

Each type of nanomembrane offers specific advantages, depending on its application, such as extending shelf life, sterilizing, reducing flavor loss, or generating eco-friendly packaging. The optimal choice depends on the nature of the food, the process requirements, and the storage conditions. Polymeric nanomembranes composed of polyamide, poly(ethylene oxide), modified chitosan, starch, cellulose, and other materials appear to be easily processable and scalable industrially. They are versatile, considering their adaptability for various applications, ranging from filtration to packaging. They have also proven compatible with active molecules, such as antioxidants or antimicrobials, and exhibit a good response to temperature and pH changes. Biodegradable types are sustainable, non-toxic, and safe for food contact, can incorporate enzymes or natural active compounds, and have an increased potential for being environmentally friendly.

Metal nanomembranes or those containing metal nanoparticles (e.g., Ag, Zn, Cu) have been shown to possess strong antimicrobial properties, which can prevent the spoilage of perishable foods. Their application could reduce the need for chemical preservatives, and they may exhibit specific properties at very low concentrations.

The porous crystalline structures of zeolite nanomembranes offer increased molecular selectivity, high chemical and thermal resistance and are ideal for applications such as dehumidification or aroma filtration. Carbon-based nanomembranes can be obtained with extraordinarily thin dimensions and are characterized by improved strength. They also exhibit high conductivity and natural antibacterial properties, enabling rapid flow and enhancing filtration efficiency.

## 3. Conclusions

Carbon nanomembranes offer advanced solutions for the food industry, improving food safety, reducing pollution, and optimizing production processes. The method of streamlining comes from the possibility of recovering valuable biomolecules from food waste or byproducts, such as whey or fruit and vegetable marc [109]. However, adequate regulation and monitoring of their impact on the environment and human health are essential to ensuring responsible and sustainable use.

Metal nanomembranes offer innovative solutions for the food industry, improving food safety, reducing contamination, and optimizing production processes. However, strict regulation of their use is essential to minimize environmental and human health risks. Although the technology is promising, further research is necessary to fully comprehend the long-term effects of metal nanoparticles on the ecosystem and the food chain.

Polymer nanomembranes are versatile and offer effective solutions for the food industry, contributing to improved food safety, extended shelf life, and reduced contamination. However, developing biodegradable and sustainable materials is important to minimize their negative environmental impact. Although their advantages are considerable, their use must be regulated to ensure food safety and protect ecosystems.

Zeolite nanomembranes are an innovative and sustainable technology with applications in the food industry, including water purification, toxin removal, active packaging, and catalysis. Their implication in enzymatic reactions can be realized by immobilizing the catalyst on the membrane-covered area [110]. They contribute to food safety, reduce contaminants, and extend the shelf life of products.

**Figure 3:**
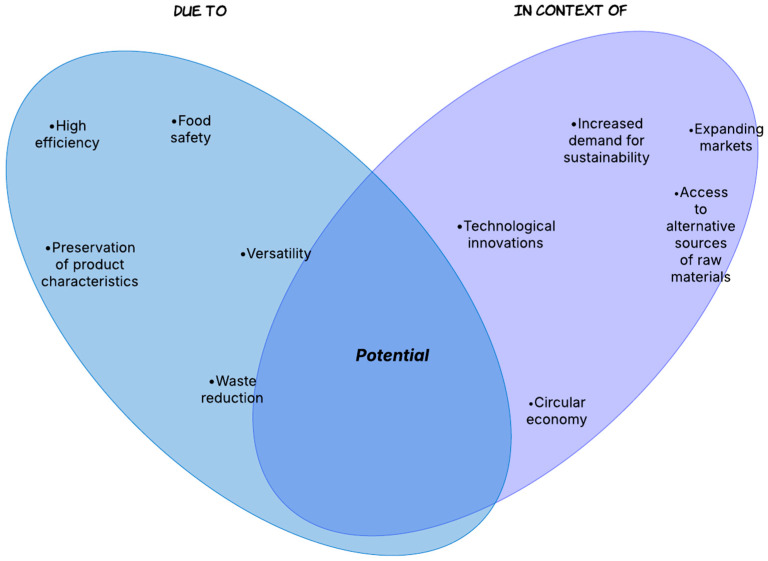
Sustainability and opportunities resulting from implementing nanomembranes in food processing.

**Figure 4:**
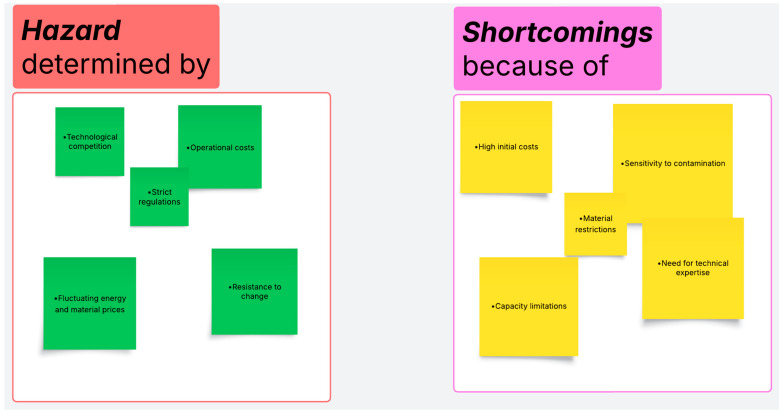
Vulnerabilities and threats due to implementing nanomembrane techniques in food processing.

However, the production cost, impact on ecosystems, and safety of their use in direct contact with food must be managed to maximize their benefits and reduce their negative environmental impacts. Figure 3 and Figure 4 schematically emphasize the strengths, feasibility, weaknesses, and constraints of applying membrane technology in the food industry.

Among the key benefits of implementing membrane techniques in food processing is their significant efficacy, which offers the opportunity to achieve precise separation and purification of target compounds with minimal negative impact on product quality. Improvements can also be achieved in terms of reduced energy consumption compared to classical thermal or chemical methods. Low-temperature processing protects thermolabile compounds such as vitamins, enzymes, and natural flavors.

As highlighted in several articles, these instruments can be utilized in various processes, including desalination, concentration, microbiological filtration, and recovery of subproducts. Their potential use for treating and recovering byproducts [111,112], such as whey or wastewater, could reduce pollution and support the circular economy [113]. These approaches respond to the growing need for regulations among many users, who increasingly recognize the importance of adopting green technologies in the food industry. The mechanism plays a significant role in reducing the carbon footprint by enhancing energy efficiency and promoting the reuse of resources. In this way, these membranes facilitate the use of alternative raw materials, such as brackish water or food waste, transforming these materials into valuable resources.

Food innocuity may result from eliminating microorganisms and harmful particles [114]. Nanomembranes possess secondary attributes beyond antibacterial activity, contributing to food preservation and functional design [115,116].

In the context of food packaging innovation, nanotechnology offers solutions for extending shelf life, improving mechanical and barrier properties, detecting contaminants, and monitoring freshness. Nanoparticle-based packaging materials, such as titanium dioxide (TiO_2_), silver, zinc oxide (ZnO), and nanoclays, are increasingly being used. However, there are legitimate concerns about these nanoparticles migrating into the food matrix, with possible toxic effects. The migration of these nanoparticles from packaging into food can occur through several mechanisms, including diffusion, which is particularly relevant for plastics, as well as desorption. In this situation, nanoparticles loosely bound to the packaging matrix can be released through mechanical or thermal contact. Other favorable conditions may be related to the deterioration of the packaging material during exposure to UV radiation, high temperatures, or changes in pH or solubility. Some compounds, such as ZnO and Ag, can release metal ions in aqueous or acidic environments, with toxicological implications.

The pro-migration factors related to the types of particles, such as their size, shape, or degree of aggregation or agglomeration, should also be taken into consideration. Other factors that need to be mentioned are the polymer matrix composition, density, and food characteristics, such as fat content, pH, storage temperature, contact time, and processing conditions (e.g., sterilization). Paidari et al. concluded that the quantity of nanoparticles migrating into the product is generally lower than the allowed amount [117]. Their study also noted the synergetic effect of a lower pH value on the tendency of silver to migrate [118]. A possible solution to minimize this migration tendency was suggested by Stormer et al., which consisted of particle encapsulation [119]. Independent of the potential factors and ways the nanoparticles could migrate into the food, specific regulations could help in the process of determining possible consumer exposure. The guidance published in the *EFSA Journal* specifies that a user is not considered vulnerable to solubility/dissolution in a marketed product or food if, at the expected maximum levels, the substance is fully dissolved in an aqueous or non-aqueous matrix or if the residues in food are below the reported/relevant solubility limit [120].

Implementing membrane-based systems involves significant investments in equipment and infrastructure. At the same time, it is worth noting that membranes can be susceptible to clogging or degradation, necessitating periodic maintenance and replacement. Considering these aspects, regularly maintaining and replacing membranes can lead to increased costs in the long term [121].

Moreover, some materials may be incompatible with certain chemicals or extreme conditions such as pH, pressure, salinity, or temperature [122], which are critical elements in food processing [123]. There is a need for technical expertise to solve such inconveniences. Additionally, the optimal operation and maintenance of membrane systems requires qualified personnel to be properly trained to ensure compliance with the regeneration parameters needed for the membrane cleaning process [124]. Another aspect that must be mentioned is that these technologies can be less efficient for very large processing volumes than conventional methods. Other emerging technologies, such as advanced filtration, may offer more efficient alternatives.

As this domain expands, legal requirements regarding the use of certain materials or waste disposal may become necessary. Such a situation could complicate the implementation of the technology. The variable costs of energy and membrane construction materials could also affect economic viability.

## 4. Discussion, Limitations, and Future Developments

Nanotechnology has revolutionized multiple fields, including the food industry, by developing nanomembranes for filtration, purification, and preservation. However, implementing these technologies raises various technical, economic, and regulatory challenges [125,126]. This study examines the current advancements in nanomembranes for the food industry, their limitations, and their potential future evolution.

Some possible limitations of such technologies relate to high costs since the production of nanomembranes involves advanced materials and expensive technologies. The increased implementation of nanomembranes will determine financial efficiency through cost reduction [127]. Although the production of membranes can be considered environmentally friendly, their commercialization is only allowable if all safety requirements are met. There could be situations where products with good technical characteristics are restricted to market placement [128]. Moreover, the investments required for their integration into food processing plants are significant. Other possible constraints may be determined according to their durability and stability. If these instruments are considered for functions characterized by high temperatures, extreme pH values, or aggressive chemicals, their efficiency and lifespan could be affected. In processes such as sterilization at ultra-high temperatures or the filtration of acidic media, which is encountered, for example, in acetic fermentation, some membranes can degrade rapidly. Another possible constraint in nanomembrane use relates to their susceptibility to (bio)fouling. This constraint is possibly influenced by the formation of biofilms (layers of microorganisms or precipitate) on the nanomembrane’s surface, reducing its efficiency.

The specific composition of the food, such as its acidity, fat, protein, and sugar content, can limit the utilization of nanomembranes. An increased sensitivity to low pH values can be observed for metal nanomembranes, such as those with silver nanoparticles, zinc oxide, or aluminum-based structures. Their use in citrus juice or canned tomato processing may induce metal corrosion, nanoparticle dissolution, reduced durability, modification of barrier properties, or the possible migration of metal ions into the food, which implies toxicity risks. Such aspects may represent a food hazard, and long-term stability tests are necessary depending on the type of food processed or packaged. Many current studies focus on the technical performance of nanomembranes under ideal conditions. There appears to be limited research on their application for a range of foods, including fermented foods, fatty foods, and alcoholic products. Integrating food science, toxicology, and materials engineering requires an interdisciplinary approach.

As the nanomembrane application domain expands to the food field for processing and production, regulations and safety measures must be developed and implemented. Although nanomembranes represent a potentially significant transformation in food sustainability, their large-scale implementation poses substantial challenges. Among the most pressing difficulties are the potential toxicity of the materials used and their high production costs, which may limit their accessibility and adoption at an industrial level.

A central concern is the safety of using nanomaterials in direct contact with food products. Certain membranes based on metal oxides (such as TiO_2_ or ZnO) or carbon materials (graphene and nanotubes) can generate toxic reactions under specific conditions, particularly when the particles are unstable or migrate into the finished product. To mitigate this risk, recent research has focused on functionalizing the surfaces of nanomembranes to prevent the release of particles and developing alternative, safer, biocompatible materials. Biodegradable polymers, such as polylactic acid (PLA), polycaprolactone (PCL), and chitosan, have emerged as viable options, offering both food safety and environmental benefits due to their degradability and renewable origins. The use of nanomembranes must be subject to strict rules regarding food safety and preservation. The factors related to possible risks to human health and the environment must be thoroughly evaluated. Special attention must be given to the component’s ecotoxicity potential [129]. Concern must be directed toward establishing the potential for molecules to migrate into the food [130,131,132], considering the temperature or pH value and their nanoscale, which might facilitate cellular crossover [133,134]. Possible interactions with other food matrix elements must also be considered [135]. Another aspect that might constrain the use of nanomembranes is their potential influence on the organoleptic properties of food. Intensive filtration may influence a food’s taste, aroma, or texture [136]. The taint susceptibility of nanomembranes, which determines the yield filtration decrease over consecutive cycles, must not be neglected [83,137].

Considering the possible limitations highlighted, there is a need to balance the potential remediation properties with the preservation of sensory quality, health, and environmental quality before implementation at an industrial scale.

Solutions for the weaknesses highlighted above could be developed through research and innovation. From this perspective, we could consider outcome optimization. This approach entails the development of more resilient nanomembranes with enhanced chemical and thermal stability.

Another significant barrier is the high cost of producing nanomembranes, particularly due to the complex manufacturing processes and expensive raw materials required. An effective strategy to reduce these costs is to move from experimental laboratory-scale production to industrial-scale production. Techniques such as continuous electrospinning, template-assisted self-assembly, and 3D printing [138,139] enable the mass production of nanomembranes with high precision at a significantly lower cost per unit. In addition, recycling and reusing membranes through chemical or thermal cleaning and regeneration [140] can extend the product’s life cycle and reduce the need for frequent replacement. Tian et al. suggested a similar aquatic permeation yield to the initial one [141]. Yang et al. also highlighted the viability of membrane heavy metal individual elimination [142]. Such particles can often be encountered along the food production chain, and their removal is necessary. The possibility of developing self-cleaning membranes supposes the integration of nanostructures with photocatalytic or hydrophobic properties. These confer the capability of self-decontamination in the presence of light or water.

Preserving the sensory profile during nanofiltration processes is essential for maintaining the quality of food products. By using membranes with precisely controlled porosity, natural or layered compositions, and optimizing processing conditions, it is possible to significantly reduce the loss of aroma, taste, and texture.

Emerging technologies, such as functionalized, stimulus-responsive, or electrospun membranes and gentle cold or inert atmosphere filtration processes, are promising for selective filtration without sensory compromise.

Based on zero-waste principles, another option is integrating biodegradable and sustainable materials. In this light, considering the second manufacturing cycle for trash could be a sustainable vision. This approach may overcome another disadvantage of new products: the need for cost optimization. Waste prices may be lower or minimal compared to first-loop raw materials. A similar result may be obtained by increasing production’s scalability for widespread use.

Since this article targets food industry applications, clarified regulations and standardization are required. In-depth studies will be conducted to assess the impact on human health to develop clear guidelines for the safety of nanomembranes.

Considering the current industrial process, nanomembranes must be adapted to advanced methods such as microfiltration and reverse osmosis. An increased yield may be obtained through synchronization with other technologies.

Although nanomembranes offer multiple advantages in the food industry, significant challenges remain related to costs, regulations, and impact on food. Nanomembranes can become a viable and sustainable solution for the future food industry by optimizing materials, reducing costs, and clarifying rules.

In the medium and long term, integrating nanomembranes into food processes could become economically and environmentally sustainable, especially if policies supporting research and development, as well as partnerships between industry and academia, are adopted, along with clear safety and performance standards. Thus, overcoming current obstacles will not only enable the widespread adoption of these technologies but also accelerate the transition of the food industry toward a greener and more efficient model. The greener perspectives brought about by using membranes in food processing also stem from the possibility of reusing filtered wastewater in various technological steps, considering the final product parameters or the separation of lipidic and aqueous fractions. Several articles underline the positive results of integrating the membrane into the technological approach. The sustainability can be further improved if the filterable area can be regenerated [143,144].

Another trend is the development of biodegradable polymeric materials. These include natural polymers such as polylactic acid, chitosan, cellulose, and pectin. Using this approach in manufacturing ensures the production of sustainable nanomembranes and/or edible products.

The future of nanomembranes in the food industry requires research to develop functional, sustainable, and safe solutions adapted to the diversity of food matrices. A priority is the composite nanomembrane design, which combines affordable polymeric materials with functional additives, such as zeolites or metal nanoparticles, to balance cost, separation performance, and structural durability. In parallel, the need to integrate these structures into active or intelligent food packaging capable of detecting and reacting to environmental changes (for example, pH, humidity, or microbiological contamination variations) is becoming increasingly clear.

Nanomembranes based on natural biopolymers, such as chitosan, alginate, denatured proteins, and nanocellulose, may be of interest due to their biodegradable properties. Biopolymers form semipermeable networks that are more selective toward polar molecules, allowing them to retain volatile compounds that contribute to flavor. The raw materials may be sourced from agro-waste valorization, which can enhance the sustainability of the process. Adding broth to food processing could have benefits, as it offers excellent food compatibility and is biodegradable, making it functionalize with enzymes.

For a safe and efficient implementation, research must be supported by a solid interdisciplinary collaboration involving fields such as food chemistry (for compatibility with food compounds), microbiology (for the validation of antimicrobial effects), food toxicology (for the assessment of substance migration in food), and materials science (for the optimization of porosity and thickness properties). Additionally, utilizing advanced computational methods, including molecular simulations and predictive models based on artificial intelligence, can significantly contribute to the rational design of nanomembranes for specific applications. Thus, progress in this field depends not only on technological innovation but also on integrating knowledge from multiple scientific areas, aiming to satisfy food safety requirements, achieve industrial efficiency, and enable environmental protection.

## Figures and Tables

**Table 1:** Application of nanomembrane techniques in food production.

Type and Components	Application Field	Target Element/Activity and Efficiency	Reference
bio-adsorbent nanomembranes graphene oxide, rosin and silver 3% nanoparticles, chitosan, and polyvinyl alcohol	water	The maximum removal efficiency at the following: temp, 30 °C; time, 120 min; ions conc. = 10 mg/L; adsorbent dose, 2 g/L. Cd^3+^, Co^2+^, Cu^2+^, Mn^2+^, Ni^2+^, Pb^2+^, Sr^2+^, and Zn^2+^ at pH 6 were 87, 84, 86, 88, 87, 96, 99.2, and 94%, while for Fe^3+^ = 92 and Cr^3+^ = 98 at pH 4, while Al^3+^ = 96 at pH 5. Inhibitory zone (mm) *E. coli* 17 ± 0.19 and *S. aureus* 19 ± 0.23.	[60]
photothermal film carbon nanoparticles 0.2%, and cellulose nanocrystals	kiwifruit	Weight loss = 2.38% after 8 days of storage	[61]
film chlorogenic acid carbon dots, and polysaccharide–alginate	apple slides	Antibacterial rates against *Escherichia coli* = 86.0% and *Staphylococcus aureus* = 91.6%	[62]
film carbon quantum dots, iron-based metal–organic framework, and agar/gelatin (blend polymers)	cherry tomatoes	Antioxidant capability of 29.1% for DPPH and 62.5% for ABTS Freshness decreases by d 1.9 ± 0.1 kg/cm^2^ after 24 days of storage	[63]
composite film nitrogen-doped carbon dots, and chitosan	blueberries pork	Photodynamic antibacterial rates for *E. coli* = 91.2% and *S. aureus* = 99.9%	[64]
pineapple leaf waste film and carbon dots 6%	pork	Antioxidant activity (IC50) against DPPH = 34.77 g/mL TVB-N 3.36 ± 0.74 mgN/100 g after 3 h of UV exposure	[65]

**Table 2:** Application of metal nanomembrane techniques in food production.

Type and Components	Application Field	Target Element/Activity and Efficiency	Reference
active packaging film gelatin, chitosan, ferulic acid, and titanium dioxide nanohybrid particles (0.40 mg/mL)	fruits preservation; strawberry and banana	antioxidant activity reported to radical scavenging activities (%) DPPH 58.99, ABTS 50.52 antimicrobial activity through relative bacteriostatic rate against (%) *E. coli* 99.51, *S. aureus* 99.80	[71]
bionanocomposite pectin, hydroxyethyl cellulose, clay, and titanium dioxide nanoparticles	wastewater	antimicrobial activity *E. coli*, *Salmonella* and *Candida* spp. total removal after 120, 30, and 60 min	[72]
composite silver nanoparticles (10–20 μg/mL) and covalent organic frameworks	green grapes	water permeability 53.94 ± 4.28% antimicrobial activity (*S. aureus*, *E. coli*, *B. subtilis*) to a bacteriostatic rate of 94.01–98.77%	[73]
nanocomposite chitosan-based silver nanoparticles	refrigerated tomatoes	inhibition zones against standard bacterial strains (mm) *S. aureus* 11.07 ± 0.76, *E. coli* 9.19 ± 0.28, *S. enterica* 9.87 ± 0.35, *L. monocytogenes* 10.12 ± 0.01, and *A. hydrophila* 9.69 ± 0.3	[74]
silk sericin-based silver nanoparticles	tomatoes	antibacterial activity reported for inhibition zones of 20 ± 0.5 mm against *Pseudomonas* sp. RTCS 2 and 16 ± 0.4 mm against *Staphylococcus* sp. weigh decrease of 15.5% after 18 days of storage	[75]
nanocomposite film silver nanoparticles, berry wax, and chitosan	rabbit meat	water vapor permeability decreased from 6.5 to 3.5, light transparency decreased from 10 to 0.78%, and opacity increased from 1.76 to 9.96% in a linear dependency on AGNP concentration from 0.5% to 1.5% *w*/*w* variation inhibition diameters with 1.25% AgNPs against *E. coli* were 15.0 mm, and 1.5% AgNPs against *S. aureus* were 13.0 mm	[76]
packaging film silver–iron nitroprusside nanoparticles and starch–xanthan gum	blueberries	fruit preservation through weight loss of 16.83% antibacterial activity inhibitory zone (mm) was *S. aureus* 21, *L. monocytogenes* 23.25 ± 0.25, and *S. enterica* 22.25 ± 0.25, and *E. coli* c 20 IC50 values against DPPH 133.7 μg/mL and ABTS 623.5 μg/mL	[77]
photosensor (light-assisted electrochemical biosensor) glassy carbon electrode-modified silver-doped titanium dioxide/Mxene nanocomposite	sensitivity	*E. coli* LOD: 1 CFU/mL Linearity 1–17 CFU/mL	[78]
nanocomposite silver nanoparticles synthesized from fruit waste grape seed extracts	grape	inhibition zone diameter (mm) *P. chrysogenum* T16 17.0 ± 1.0 and A. niger ATCC16404 17.8 ± 0.3 weight loss after five storage days of 45%	[79]
nanocomposite poly(vinyl alcohol), agar, maltodextrin, and silver nanoparticles 6%	banana	antibacterial inhibition zone at 19 mm against *E. coli* and 15 mm for *S. aureus* respiration rate (mgCO_2_/kg/h) on day 5 was 105.30 ± 1.93; and 55% lower weight loss compared to the uncoated fruit	[80]

**Table 3:** Application of polymer nanomembrane technique in nourishment production.

Type and Components	Application Field	Target Element/Activity and Efficiency	Reference
matrix milk fat globule membrane, and pullulan polysaccharide	encapsulation	increased viability after storage at different temperatures of *Lacticaseibacillus rhamnosus*; GG	[91]
Bionanocomposite with 0.4 wt% pracaxi oil nanoemulsion and plasticized xylitol–pectin matrix	butter stability	water permeability insurance after 60 days, reported to the malondialdehyde-formed level DPPH antioxidant activity was 36.3 ± 0.3%	[92]
film chitosan, polyvinyl alcohol, and shikonin from *radix* Lithospermi, 2%	shrimp freshness	antimicrobial activities reported at the inhibition area (mm) for *Escherichia* and *Staphylococcus aureus* were 2 ± 0.00 and 8 ± 0.02 antioxidant activity radical inhibition (%) DPPH 98 ABTS 67	[93]
film poly(N-isopropylacrylamide), polyvinyl alcohol, polylactic acid, and lemon essential oil	extended blackberry shelf life	average weight loss of 0.6 (%) average firmness of 6.72 (N) average inhibition rate initially for antibacterial activity (%) *Escherichia coli* ≈ 45 *Staphylococcus aureus* ≈ 48	[94]

## Data Availability

The original contributions presented in this study are included in the article. Further inquiries can be directed to the corresponding author.
